# Does intraoperative mechanical prophylaxis prevent venous thromboembolism in total knee arthroplasty? – effectiveness of passive-assisted ankle motion in surgical/non-surgical side

**DOI:** 10.1186/s42836-021-00088-2

**Published:** 2021-09-03

**Authors:** Toshiyuki Tateiwa, Tsunehito Ishida, Toshinori Masaoka, Takaaki Shishido, Yasuhito Takahashi, Jun Nishida, Kengo Yamamoto

**Affiliations:** 1 Department of Orthopedic Surgery, Tokyo Medical University, 6-7-1, Nishishinjuku, Shinjuku-ku, 160-0023 Tokyo, Japan; 2 Department of Bone and Joint Biomaterial Research, Tokyo Medical University, 6-7- 1,Nishishinjuku, Shinjuku-ku, 160-0023 Tokyo, Japan

**Keywords:** Total knee arthroplasty, Deep venous thrombosis, Mechanical prophylaxis, Passive-assisted ankle motion

## Abstract

**Background:**

Gradual compression stocking (GCS) and intermittent pneumatic compression device (IPCD) are used for intraoperative mechanical prophylaxis against venous thromboembolism (VTE) during total knee arthroplasty (TKA). In this study, we applied a passive-assisted ankle motion in combination with GCS and IPCD during TKA and evaluated its effectiveness in preventing postoperative VTE.

**Methods:**

We included 77 patients who underwent primary unilateral TKA. Patients were divided into group A (53 patients who underwent GCS and IPCD on their non-surgical side limb) and group B (24 patients who underwent passive ankle dorsiflexion motion in addition to GCS and IPCD on their non-surgical side limb). Deep vein thrombosis (DVT) was assessed using lower extremity ultrasonography (US). The incidence of VTE in each affected limb was compared between the two groups.

**Results:**

US was performed 4 days after surgery on average. The incidence of DVT in groups A and B was 47.2 and 70.8 %, respectively. In group A, 22.6 % of DVTs were found only on the surgical side, 11.3 % on the non-surgical side, and 13.2 % on both sides. On the other hand, in group B, 41.7 % of DVTs were found only on the surgical side, 4.2 % on the non-surgical side, and 25.0 % on both sides. No significant difference in the incidence of VTE was noted between the 2 groups.

**Conclusions:**

The intraoperative application of passive ankle motion plus GCS and IPCD might not further reduce the incidence of postoperative DVT in TKA patients.

## Introduction

A high risk of venous thromboembolism (VTE) after total knee arthroplasty (TKA) has long been recognized in the field of orthopedics. The 8th edition of the American College of Chest Physicians (ACCP) guidelines reported that the incidence of overall and proximal VTE without prophylaxis was 41–85 % and 5–22 %, respectively [1], while the Japanese Orthopaedic Association (JOA) reported incidences of 50–60 % and 9–16 %, respectively [2]. Since the importance of prophylaxis against VTE has been appreciated, several different methods are currently used, including pharmacological (*e*.*g*., a low-molecular-weight heparin [LMWH] or factor X inhibitor) and mechanical (*e*.*g*., a gradual compression stocking [GCS] and/or intermittent pneumatic compression device [IPCD]) prophylaxis. Despite the widespread application of these methods, the incidence of VTE following TKA remains relatively high. In recent years, pharmacological prophylaxis has become popular, and several authors have demonstrated their efficacy and safety as prophylactic measures against VTE [3–6]. Nevertheless, serious hemorrhagic adverse events were reported to be associated with their use [3, 7, 8]. In the 8th edition of the ACCP guidelines, pharmacological prophylaxis had been listed as Grade 1 A recommendation [1], but its recommendation was subsequently down-graded in the 9th edition (Grade 1B recommendation) [9]. In contrast, since mechanical prophylaxis is a safer option, its effectiveness has been re-considered in recent years [10–12] and its recommendation was upgraded (Grade 1 C recommendation) [9].

In response to the aforementioned developments, we modified our thromboprophylactic strategy by introducing intraoperative passive ankle motion plus GCS and IPCD. The purpose of the present study was to investigate the effect of intraoperative passive ankle motion on the incidence of VTE after TKA.

## Patients and methods

This study was approved by the institutional review board (IRB) and the corresponding ethics committee, and written approval statements were obtained from all patients before surgery.

**Fig. 1:**
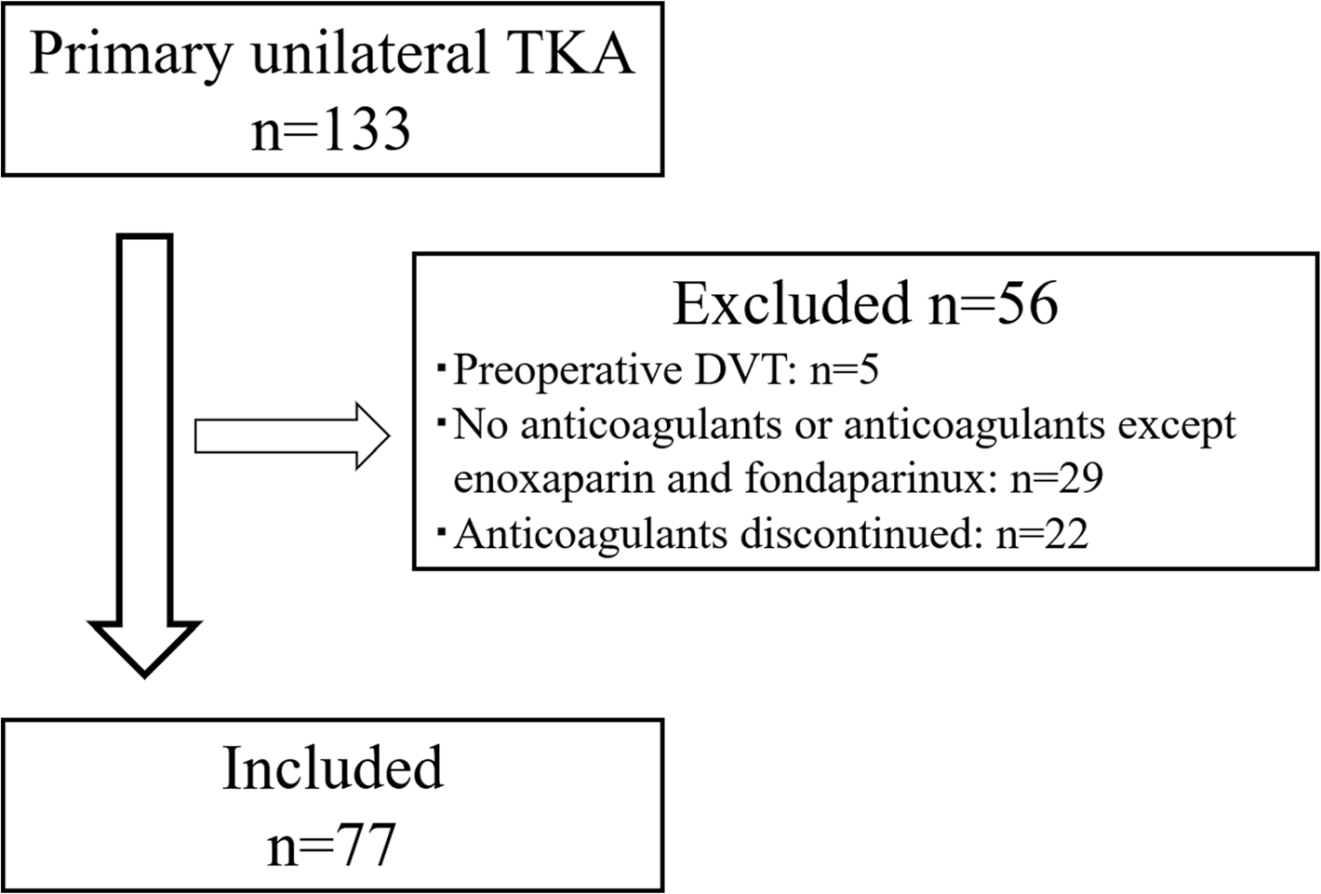
Flow diagram of patients analyzed in this study

A total of 133 patients from our institute who had undergone primary unilateral TKA between January 2011 and August 2014 were recruited into the present study (Fig. 1). The inclusion criteria were: patients had no preoperative DVT, and were continuously administered anticoagulants (either enoxaparin or fondaparinux) until DVT was detected by lower-extremity ultrasonography (US) within the postoperative (P.O) day 8. Five patients with preoperative DVT as seen on US examination were excluded. Eighteen patients who had not been given any anticoagulants were excluded. Eleven patients who had received anticoagulants except enoxaparin and fondaparinux were excluded. Twenty-two patients in whom anticoagulants were discontinued prior to US examination were excluded. A total of 77 TKA patients (14 men and 63 women) were analyzed in this study. At the time of surgery, patients’ mean (mean ± SD) age and body mass index (BMI) were 73.0 ± 8.6 years (range, 50–87 years) and 25.8 ± 3.6 kg/m^2^ (range, 17.3–37.1 kg/m^2^), respectively. The primary diseases were osteoarthritis in 68 patients, rheumatoid arthritis in 7, and necrosis of the femoral condyle in 2.

**Fig. 2:**
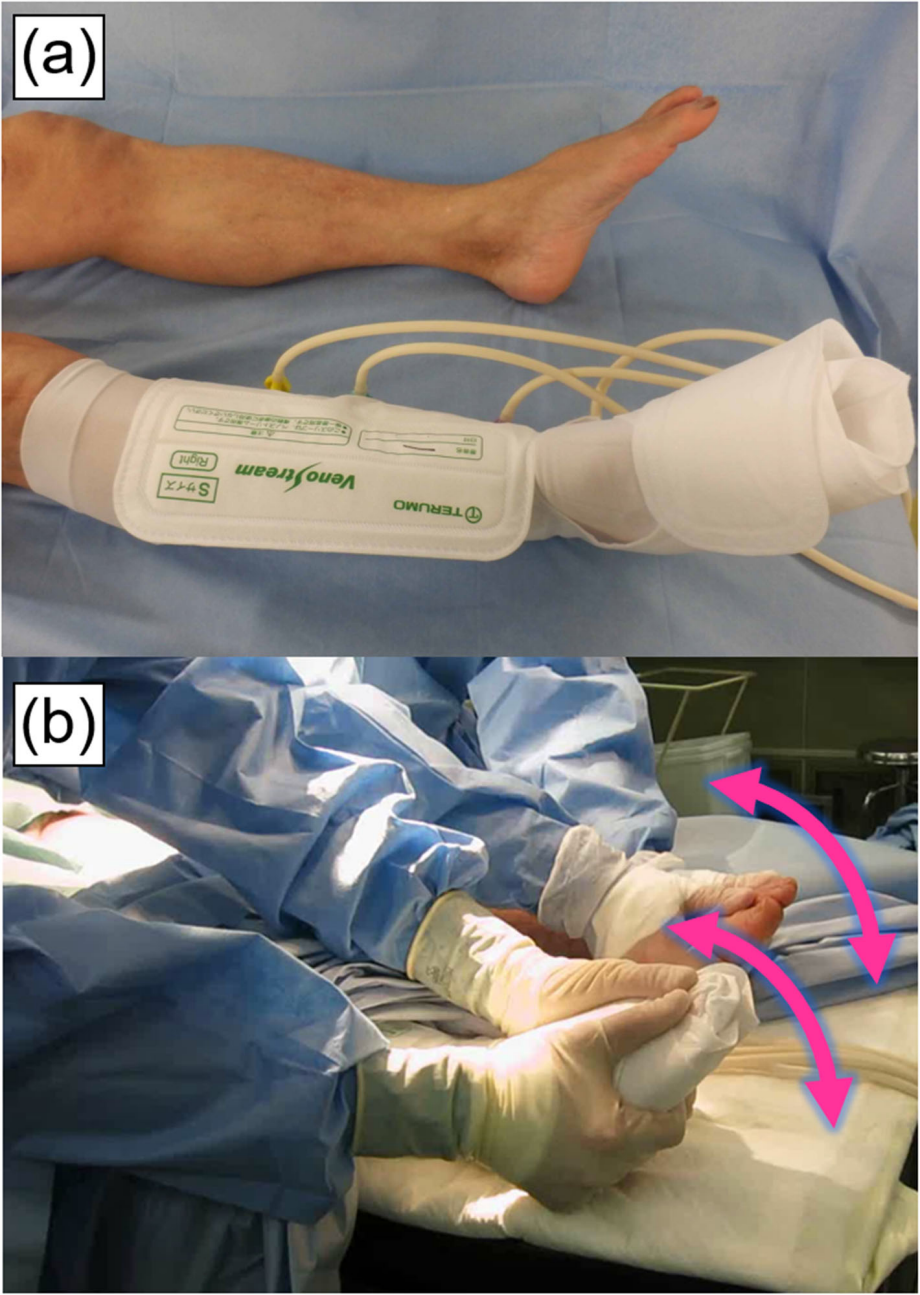
Photographs of a gradual compression stocking (GCS) and intermittent pneumatic compression device (IPCD) (**a**) and intraoperative passive plantar flexion motion (**b**)

General and spinal anesthesia was performed in 71 and 6 patients, respectively. In all cases, TKA was performed via the mid-vastus approach, with a tourniquet used. The Scorpio® NRG knee system (Stryker Inc., Mahwah, NJ, USA) was employed in all patients. Cruciate-retaining (CR) and posterior stabilizer (PS) type implants were inserted into 71, and 6 patients, respectively. All components were fixed with cement. GCS and IPCD (Veno Stream®R, Terumo, Tokyo, Japan) with a pressure of 60 mmHg were applied to the non-operated limb (Fig. 2a), and a drain was placed postoperatively. As an intraoperative mechanical prophylaxis, passive plantar flexion motion (Fig. 2b) was applied simultaneously to both sides of the ankle joint for up to 100 times in total at a rate of 3 times/sec. The procedure was continuously performed by two medical staff before the application of air tourniquet and during the first and second release of the tourniquet (*i*.*e*., 300 [100 × 3] times in total). The first tourniquet release before cementing implants was performed for detecting any arterial branch injury. The mean duration of tourniquet inflation was 50.3 and 38.4 min, respectively (88.7 min in total).

Pulse Doppler, color Doppler, and compression US examinations were performed using the Xario™ XG (Toshiba Medical Systems, Tokyo, Japan) with 7.5-MHz linear and 3.5-MHz convex probes by clinical vascular technologists to monitor the development of DVT within the first 7 days after surgery (Xario™ XG, Toshiba Medical Systems). Distal DVT was defined as DVT localized to the veins of the lower legs (*i*.*e*., soleal vein, peroneal vein, tibial vein, gastrocnemius vein), and proximal DVT as DVT occurrs in the region proximal to the popliteal vein. Contrast-enhanced computed tomography was performed to screen for pulmonary embolism (PE) in patients with proximal DVT detected on US, chest symptoms, or decreased arterial oxygen saturation (SaO_2_).

A venous foot pump (VF) (A-V Impulse™, Covidien, MN, USA) was applied after surgery, and the drain was removed on postoperative (P.O.) day 2. Joint range-of-motion exercise was started by using a continuous passive motion device and ambulation was started with a walker. VF was continued until stable ambulation was achieved and GCS was used for 2 weeks after the removal of VF. Thromboprophylaxis with fondaparinux at 2.5 mg/day or enoxaparin at 4000 IU/d was started on P.O. day 2 after the drain removal and continued for 7 days (until P.O. day 8). Nevertheless, when DVT was detected using US, enoxaparin and fondaparinux were discontinued and switched to heparin or warfarin. Administration of the latter drugs was continued until resolution of DVT or for 3 months after surgery.

For analysis, patients were divided into two groups (Table 1): group A (53 patients who had undergone GCS and IPCD on their non-surgical side limb in primary unilateral TKA between January 2011 and March 2013) and group B (24 TKA patients who had undergone passive ankle dorsiflexion motion plus GCS and IPCD on their non-surgical side limb in primary unilateral TKA between September 2013 and August 2014). The incidence of VTE and the affected limbs were compared between the two groups.

**Table 1:** Comparison of the patient characteristics between groups A and B

	Group A (*n* = 53)	Group B (*n* = 24)	*P*-value
Age (years: mean ± SD)	73.2 ± 8.3	72.5 ± 9.5	0.77
Sex (men: women)	9 :44 (17.0 %: 83.0 %)	5: 19 (20.8 %: 79.2 %)	0.75
BMI (kg/m^2^: mean ± SD)	25.6 ± 3.4	26.1 ± 4.2	0.62
Operation time (min: mean ± SD)	96.8 ± 15.7	92.5 ± 20.0	0.30
Total tourniquet time (min: mean ± SD)	90.1 ± 14.6	85.8 ± 19.2	0.29
Thromboprophylaxis (enoxaparin:fondaparinux)	49:4 (92.5 %: 7.5 %)	23:1 (95.8 %: 4.2 %)	1.00

Two-tailed Student’s *t*-test and chi-square test were performed using Graphpad Prism software, version 6.05 (GraphPad Software, Inc., San Diego, CA) to test for statistically significant differences between groups A and B. In addition, a chi-square test was used to determine whether a relationship existed between US examination day and detection rate of DVT. The power of relationship in chi-square test was measured in terms of Cramer’s V, with the effect being deemed small when V was at 0.10, medium at 0.3, and large at 0.5 [13]. Statistical difference in comparison was considered to be statistically significant when a *P* < 0.05.

## Results

The US examination was performed at a mean time of 3.9 ± 1.5 days after surgery. It should be noted that there was no significant association between US examination days and detection rate of DVT (chi-square = 4.525, *P* = 0.48, Cramer’s V = 0.24) (Table 2). The incidences of the overall, distal, and proximal DVT were 54.5 %, 53.2 %, and 1.3 %, respectively (Table 3). As to VTE, 16.9 % affected in both limbs, 28.6 % only involved the surgical side, and 9.1 % developed only on the non-surgical side. 1.3 % of patients had asymptomatic PE. Two patients with proximal DVT and one with decreased SaO_2_ received contrast-enhanced computed tomography for detailed examination for PE and one patient with proximal DVT had asymptomatic PE.

**Table 2:** The number of patients in whom DVT was detected and not detected on US performed in different postoperative days

Postoperative day	DVT	
	Present	Absent
2	5	1
3	23	19
4	1	2
5	4	3
6	6	9
7	3	1

Note: No significant association between the DVT examination day and its detection rate (chi-square = 4.525, *P* = 0.48, Cramer’s *V* = 0.24)

**Table 3:** The prevalence of postoperative DVT in TKA patients

	Prevalence (%) (number of patients [knees])
Distal DVT	53.2 (41/77)
Proximal DVT	1.3 (1/77)
Bilateral DVT	16.9 (13/77)
Surgical side DVT	28.6 (22/77)
Non-surgical side DVT	9.1 (7/77)

Patients in group A underwent US examination at a mean time of 4.1 ± 1.4 days after surgery, the incidences of overall, distal, and proximal DVT were 47.2 %, 45.3 %, and 1.9 %, respectively (Table 4). 1.9 % of the patients had asymptomatic PE. Patients in group B received US at a mean time of 3.6 ± 1.6 days after surgery, the incidence of DVT was 70.8 % (the distal type). There was no significant difference in the incidence of VTE between group A and group B (chi-square = 3.731, *P* = 0.053, Cramer’s V = 0.22). In group A, 22.6 % of DVTs were found only on the surgical side, 11.3 % on the non-surgical side, and 13.2 % on both sides. On the other hand, in group B, 41.7 % of DVT was found only on the surgical side, 4.2 % on the non-surgical side, and 25.0 % on both sides. The incidences of DVT in the non-operated limbs (including bilateral DVT) were 24.5 and 29.2 % in group A and group B and the difference was not significant (chi-square = 0.1848, *P* = 0.67, Cramer’s V = 0.05).

**Table 4:** Comparison of the incidence of DVT between groups A and B

		Group A (*n* = 53)	Group B (*n* = 24)	*P*-value
Postoperative US examination (days)		4.1 ± 1.4	3.6 ± 1.6	0.16
Prevalence (%) (number of patients [knees])	Distal DVT	45.3 (24/53)	70.8 (17/24)	0.05
	Proximal DVT	1.9 (1/53)	0 (0/24)	1.00
	Bilateral DVT	13.2 (7/53)	25.0 (6/24)	0.21
	Surgical-side DVT	22.6 (12/53)	41.7 (10/24)	0.11
	Non-surgical-side DVT	11.3 (6/53)	4.2 (1/24)	0.42

## Discussion

During TKA, we employed a passive-assisted ankle motion plus GCS and IPCD, as a thromboprophylactic strategy. Nevertheless, our results showed that intraoperative passive-assisted ankle motion might exert no significant influence on VTE prevention.

It was previously reported that 47.3 % of TKA patients who underwent GCS and IPCD as the intraoperative mechanical prophylaxis developed deep vein thrombosis (DVT) in the non-operated limb [14]. Ishii et al [15] investigated the velocity of blood flow in the femoral vein under resting conditions and during different mechanical therapies. They reported that the velocity was increased by 6.33 times during active ankle motion, 3.99 times during passive ankle motion, and 3.88 times during lower leg compression. Fuchs et al [16] reported that the incidence of DVT was significantly reduced from 25% to 3.6 % when non-fractionated heparin and an Arthroflow device (a passive ankle motion device) were used for prophylaxis against VTE. Furthermore, Funayama et al [17] reported that the incidence of VTE after THA was 36.9 % in patients without any prophylactic measures taken, 15.6 % in those who underwent GCS and IPCD, and 1.0 % in those who received an intraoperative manual lower-leg massage and passive ankle motion. These results highlighted the significant effect of mechanical prophylaxis, especially intraoperative manual massage and ankle motion.

In the present study, a high overall incidence of VTE (54.5 %) was noted despite the application of mechanical prophylaxis. A recent meta-analysis on lower-limb DVT diagnosis showed that mixing compression and color/Doppler technique (also used in the present study) had a greater sensitivity than pure color/Doppler technique as well as pure compression technique [18]. Apart from the methodology, we speculated that an exhaustive screening (an examination lasting over 45 min and performed by very-experienced vascular technologists) might have contributed to our high overall detection.

One prior study [14] reported that the incidence of DVT following TKA was high (47.3 %) in patients who underwent GCS and IPCD, suggesting that these methods alone have limited effectiveness against VTE. Thus, intraoperative passive ankle motion was performed in addition to GCS and IPCD in the present study. However, no significant preventive effect on DVT was observed, and the incidence of DVT remained high in the non-operated limbs.

Regarding factors underlying the high incidence of DVT, even in the non-operated limb, because the soleus muscle is an antigravity muscle, muscle pumping is ineffective in recumbent patients and blood flow becomes congested. In addition, general anesthesia, which is considered a factor for blood stagnation, may also affect the onset of DVT. Furthermore, the concomitant use of GCS and IPCD may restrict the contraction of the soleus muscle.

These findings potentially inform us that, to reduce the incidence of DVT, further intraoperative prophylactic measures, such as a lower leg massage should be considered. In addition, the first few postoperative days are particularly important from the viewpoint of DVT prophylaxis because the formation of thrombi occur predominantly from immediately after surgery to US examination. In this context, further consideration should be given to how to apply effective prophylaxis during the aforementioned postoperative period, *e*.*g*., to continue ankle motion with minimal pain or to initiate weight-bearing ambulation as early as possible to activate the pumping action of the soleus muscle.

This study had some limitations. First, because US examination was not performed during TKA, the possibility and extent of intraoperative DVT formation was not precisely known. Second, the precise onset of DVT was also unknown during the period from immediately after surgery to the US examination. However, there was no significant association between US examination day and detection rate of DVT (Table 1). Third, either enoxaparin or fondaparinux was used in our patients. However, several studies documented no significant differences in the incidence of VTE between enoxaparin and fondaparinux [19–24]. Fourth, care should be taken in generalizing our reported high incidence of postoperative DVT in TKA patients because exhaustive US examination was necessary in the present study to explore in detail the real effectiveness of intraoperative mechanical prophylaxis against DVT. Despite the above-mentioned limitations, our results indicated that the intraoperative application of passive ankle motion in addition to GCS and IPCD might not be effective in further reducing the incidence of postoperative DVT in TKA patients.

## Conclusions

We applied a passive-assisted ankle motion in combination with GCS and IPCD during TKA and evaluated its effectiveness in preventing postoperative VTE. The intraoperative application of passive ankle motion in addition to GCS and IPCD might not be effective in further reducing the incidence of postoperative DVT in TKA patients. Further research effort should be directed at how to effectively use prophylactic measures during the above postoperative period.

## Data Availability

All data analyzed during this study are included in this article.

## Abbreviations

GCS: Gradual compression stocking
IPCD: Intermittent pneumatic compression device
VTE: Venous thromboembolism
TKA: Total knee arthroplasty
DVT: Deep vein thrombosis
US: Ultrasonography
ACCP: American College of Chest Physicians
JOA: Japanese Orthopaedic Association
LMWH: Low-molecular-weight heparin
IRB: Institutional review board
CR: Cruciate-retaining
PS: Posterior stabilizer
PE: Pulmonary embolism
SaO_2_: Arterial oxygen saturation
VF: Venous foot pump
P.O: Postoperative
